# Pre-Pregnancy Diet and Vaginal Environment in Caucasian Pregnant Women: An Exploratory Study

**DOI:** 10.3389/fmolb.2021.702370

**Published:** 2021-07-28

**Authors:** Margherita Dall’Asta, Luca Laghi, Sara Morselli, Maria Carla Re, Sara Zagonari, Giulia Patuelli, Claudio Foschi, Maria Federica Pedna, Vittorio Sambri, Antonella Marangoni, Francesca Danesi

**Affiliations:** ^1^ Department of Animal Science, Food and Nutrition (DIANA), Università Cattolica Del Sacro Cuore, Piacenza, Italy; ^2^ Centre of Foodomics, Department of Agricultural and Food Sciences (DISTAL), University of Bologna, Cesena, Italy; ^3^ Interdepartmental Centre for Agri-Food Industrial Research (CIRI Agrifood), University of Bologna, Cesena, Italy; ^4^ Unit of Microbiology (DIMES), University of Bologna, Bologna, Italy; ^5^ Family Advisory Health Centres, Ravenna, Italy; ^6^ Unit of Microbiology, Greater Romagna Hub Laboratory, Cesena, Italy; ^7^ Human Nutrition Unit, Department of Agricultural and Food Sciences (DISTAL), University of Bologna, Cesena, Italy

**Keywords:** vaginal microbiome, vaginal metabolome, diet, nutrient intake, pregnancy, women’s health

## Abstract

Vaginal microbes and their metabolic products have crucial functions, affecting local immunity development and maternal-fetal health. The composition of the vaginal microbiome can vary in response to various factors, including body mass index (BMI), and diet. In this study we get new insights into the vaginal ecosystem of Caucasian women (n = 24) at the first trimester of pregnancy, assessing whether pre-pregnancy diet can affect the structure of the vaginal environment in terms of bacterial composition and vaginal metabolite concentration. We characterized 1) the vaginal bacterial composition (Nugent score), 2) the vaginal metabolic profiles (^1^H-NMR spectroscopy), and 3) the dietary nutrient intake by means of a validated food frequency questionnaire. Pre-pregnancy BMI was negatively related to vaginal health status, indicating that women who begin pregnancy overweight/obese have a greater occurrence of vaginal dysbiosis during pregnancy. A lactobacilli-dominated vaginal microbiota was negatively associated with higher pre-pregnancy intake of animal-sourced protein. Conversely, a higher pre-pregnancy consumption of total carbohydrates and sugars seemed to be a protective factor for vaginal health. The vaginal environment of BV-women was characterized by higher levels of biogenic amines and organic acids, whereas higher levels of phenylpropionate and diverse amino acids were fingerprints of a healthy vaginal status. A significant association between a higher pre-pregnancy BMI and several dysbiosis-related vaginal metabolites was also found. Our study shed light on the role of pre-pregnancy BMI and diet on the vaginal environment during pregnancy, underlining the importance of limiting protein intake from animal foods to maintain a healthy lactobacilli-dominated microbiota.

## Introduction

The cervicovaginal environment is composed by diverse microorganisms, creating dynamic and complex relationships with each other and with the host. Microbes and their metabolic products have an important role in maintaining vaginal eubiosis: they can protect women from several infections, affect local immunity development and have an impact on maternal-fetal health (Oliver et al., 2020).

In healthy reproductive-aged women, the vaginal microbiome is characterized by low bacterial diversity, being often dominated by different species of *Lactobacillus* genus (Smith and Ravel, 2017; Ceccarani et al., 2019).

However, when a deep change occurs, a dysbiosis condition called bacterial vaginosis (BV) may arise. BV is characterized by a depletion of lactobacilli, together with the growth of complex polymicrobial community of anaerobic bacteria, as *Gardnerella vaginalis*, *Atopobium* spp., and *Prevotella* spp. (Srinivasan et al., 2015). The shift in bacterial communities is associated with alterations in the vaginal metabolic profiles. High concentrations of biogenic amines (e.g., putrescine, cadaverine, and trimethylamine) and short-chain fatty acids (SCFAs, especially acetate and succinate) with low levels of some amino acids (tyrosine, glutamate) are the most common metabolic fingerprints of BV (Parolin et al., 2018; Srinivasan et al., 2015; Vitali et al., 2015).

The composition of the vaginal microbiome is influenced by various local and systemic factors, such as hormonal levels, pregnancy, smoking, sexual habits, the use of topical products or antibiotics, and the presence of urogenital infections (Kroon et al., 2018; Noyes et al., 2018; Parolin et al., 2018).

In this context, it has been shown that also anthropometric parameters (e.g., body mass index-BMI), as well as dietary habits, can impact the bacterial composition of the vaginal environment (Brookheart et al., 2019; Song et al., 2020).

Obese and overweight women have a great occurrence of bacterial vaginosis compared to lean women, thus suggesting that obesity can favor the onset of BV through different mechanisms including alterations in hormonal, metabolic, or immunological functions (Brookheart et al., 2019).

Increased dietary fat intake, energy intake and glycemic load are associated with a higher risk of BV, whereas the increased intake of folate, vitamin A, and calcium seems protective factors against BV condition (Neggers et al., 2007; Thoma et al., 2011).

To the best of our knowledge, there is no exhaustive data about the impact of pre-pregnancy diet on the composition of the vaginal environment during pregnancy in Caucasian women.

Previous works have mainly investigated the association between dietary intake and the composition of the vaginal microbiome of non-pregnant American and Afro-American women (Neggers et al., 2007; Tohill et al., 2007; Thoma et al., 2011; Tuddenham et al., 2019).

Therefore, the aim of this study was to get new insights into the vaginal ecosystem of Caucasian women at the first trimester of pregnancy, assessing whether pre-pregnancy nutrient intake and the adherence to the Mediterranean diet (MD) can affect the structure of the vaginal environment in terms of bacterial composition and vaginal metabolite concentration. For each woman, we characterized 1) the vaginal bacterial composition (microscopic scoring system), 2) the vaginal metabolic profiles (^1^H-NMR spectroscopy), 3) the nutrient intake adequacy by means of a validated method for collecting dietary data, and 4) the MD adherence assessed through a validated score.

## Materials and Methods

### Study Group and Sample Collection

From November 2019, all the Caucasian pregnant women attending the Family Advisory Health Centers of Ravenna (Italy) for prenatal care were considered eligible for the study.

Exclusion criteria were the following: 1) age <18 years; 2) HIV status; 3) medically assisted procreation; 4) use of any antimicrobial in the past month; 5) use of vaginal douches or topical agents in the previous 2 weeks; 6) presence of uncontrolled chronic diseases (e.g., diabetes, autoimmune disorders, malignancies); 7) drug addiction or heavy smokers (>15 cigarettes/day). Moreover, women with sexually transmitted infections (STIs) (i.e., *Chlamydia trachomatis*, *Neisseria gonorrhoeae*, *Trichomonas vaginalis*, *Mycoplasma genitalium*), aerobic vaginitis or symptomatic candidiasis were further excluded after laboratory testing.

During the routine clinical visit at the first trimester of pregnancy (gestational ages 9–13 weeks), demographic, anthropometric, and clinical data were recorded from each patient.

From each woman, two vaginal swabs were collected aseptically from the midpoint of the vagina. The swabs were gently rubbed for ∼20 s against the mid vaginal wall. The first one (E-swab, Copan, Brescia, Italy) was used for microbiological tests, whereas the second was collected with a sterile cotton bud, re-suspended in 1 ml of sterile saline, and stored at −80°C until use. Frozen vaginal swabs were thawed, vortexed for 1 min and the liquid was centrifuged at 10,000 × g for 15 min. Cell-free supernatants were employed for metabolomic analysis, as described below.

All subjects gave written informed consent prior to the study starting, and the protocol was approved by the Ethics Committee of Romagna (CEROM) (n°2032 of 21st February 2018).

The required sample size of the study was evaluated according to the formula proposed by Viechtbauer et al. (2015) for pilot studies. Previous works on BV in pregnant Caucasian women have demonstrated an incidence ranging from 11.6% (Hay et al., 1994) to 14% (Freitas et al., 2017). Assuming a mean incidence of 12% in our study group, we determined that an enrollment of 23 pregnant women would be sufficient to give 95% confidence to detect BV-affected women.

### Microbiological Investigations

The presence of STIs (i.e., *C. trachomatis*, *N. gonorrhoeae*, *T. vaginalis*, and *M. genitalium*) was excluded by means of a commercial NAAT (Seeplex STI Master Panel 1; Seegene, Seoul, KR), whereas microscopic examination and cultures were used for candidiasis and aerobic vaginitis diagnosis (Donders et al., 2011; Yano et al., 2019).

A microscopic Gram stain scoring system (Nugent score), based on the presence of different bacterial morphotypes (*Lactobacillus* spp., *Gardnerella vaginalis*, and *Mobiluncus* spp.), was used to assess the composition of the vaginal microbiome (Nugent et al., 1991). Women were stratified based on Nugent score, considering that an abundance of *Lactobacillus* morphotypes, yields a low Nugent score (i.e., normal lactobacilli-dominated flora) while the presence of Gram-variable small (*G. vaginalis*) and/or curved rods (*Mobiluncus* spp.) yields a high Nugent score, indicating a condition of dysbiosis/BV (Zozaya-Hinchliffe et al., 2010).

### Metabolomic Analysis

Metabolomic analysis was performed by means of a ^1^H-NMR spectroscopy. One hundred μL of a D_2_O solution of 3-(trimethylsilyl)-propionic-2,2,3,3-d4 acid sodium salt (TSP) 10 mM set to pH 7.0 were added to 700 µL of the cell-free supernatants of the vaginal swabs.

^1^H-NMR spectra were recorded at 298 K with an AVANCE III spectrometer (Bruker, Milan, Italy) operating at a frequency of 600.13  MHz, equipped with Topspin software (Ver. 3.5) (Ventrella et al., 2016; Foschi et al., 2018).

The signals originating from large molecules were suppressed by a CPMG filter of 400 spin-echo periods, generated by 180°pulses of 24 μs separated by 400 μs (Ventrella et al., 2016).

To each spectrum, line broadening (0.3 Hz) and phase adjustment were applied by Topspin software, while any further spectra processing, molecules quantification and data mining step were performed in R computational language (R: A Language and Environment for Statistical Computing, R version 4.0.5) by means of scripts developed in house.

The spectra were aligned towards the TSP signal, set at −0.017 ppm in agreement with Chenomx software data bank (version 8.3, Chenomx Inc., Edmonton, Alberta, Canada). The spectra were then baseline-adjusted by means of peak detection according to the “rolling ball” principle (Kneen and Annegarn, 1996) implemented in the “baseline” R package (Liland et al., 2010).

The signals were assigned by comparing their chemical shift and multiplicity with Chenomx software data bank. Molecules were quantified in the first sample acquired by employing the added TSP as an internal standard.

To compensate for differences in sample amount, any other sample was then normalized to such sample by means of probabilistic quotient normalization (Dieterle et al., 2006). Integration of the signals was performed for each molecule by means of rectangular integration.

### Anthropometric Measurements and Dietary Assessment

Body weight (BW) and height were self-reported at recruitment. BMI was calculated as weight (kg)/height (m^2^) and categorized according to the World Health Organization’s cut-points (WHO, 2000) for underweight (<18.5 kg/m^2^), normal weight (18.5–24.99 kg/m^2^), overweight (25–29.99 kg/m^2^) or obesity (≥30 kg/m^2^).

To assess long-term nutritional habits (over a 1-year period), participants were asked to complete a food frequency questionnaire (FFQ) developed in the European Prospective Investigation into Cancer and Nutrition (EPIC) study (Bingham et al., 2001) and validated in the Italian population (Pala et al., 2003). The FFQs were administered by a trained scientist.

The presence of mis-reporters was assessed by evaluating the ratio of reported energy intake to estimated basal metabolic rate according to the protocol developed by the European Food Safety Authority (EFSA) (Ambrus et al., 2013). All subjects resulted plausible reporters.

Individual intakes of nutrients were compared with current dietary reference values (DRVs) for macronutrients, minerals, and vitamins (WHO/FAO, 2003; FAO, 2010; EFSA, 2017) (Supplementary Table S1). In addition, moderate alcohol drinking (one drink or less in a day) (USDA, 2015) was considered as an acceptable intake.

To assess overall diet quality, the collected dietary data were used to compute the MD adherence score (MEDI-LITE) (Sofi et al., 2017). The MEDI-LITE, ranging from 0 (minimal adherence) to 18 (maximal adherence), includes food and nutrient indicators of diet quality, such as nine components focusing on the consumption of whole grains, legumes, fruit, vegetables, nuts, and olive oil (positive points), dairy, red and processed meat (negative points), and alcohol (points according to the consumption).

### Data Analysis and Statistics

The distribution of clinical parameters was evaluated using the D’Agostino-Pearson test. Student’s t-test for normally distributed data and Mann-Whitney *U* test for non-normally distributed data were used to compare the dietary intakes of the study population to the reference values. For total fat and total carbohydrates, reference intake (RI) is a range given as a percentage of total energy intake (EFSA, 2017) and the 50th percentile of the RIs was used to compare with each individual intake.

χ^2^ test was used to test for differences in BMI and MEDI-LITE score between the age groups 20–29 and 30–39.

To find correlations between the vaginal microbiota composition and nutrient intake, Nugent score (0–10) was related to anthropometric/dietary data. Correlations were searched by calculating Spearman correlation coefficient (r) after correction for multiple comparisons (i.e., Bonferroni-Holm correction). A *p*-value < 0.05 was considered statistically significant.

Metabolomic data were analyzed with R computational language (ver. 4.0.5). Vaginal metabolite concentrations were correlated to clinical (i.e., vaginal health), anthropometric (i.e., BMI), and dietary data (i.e., energy and nutrient intake, MEDI-LITE score). Trends encompassing the overall metabolome were highlighted with principal component analysis (PCA) models. To reduce influences of potential outliers, this was done by employing its robust version (rPCA) according to Hubert et al. (2005). Correlation between each molecule’s importance over principal components and its concentration were assessed according to Pearson. Raw metabolomic data are available as a Supplementary Material.

## Results

### Study Population

A total of 24 Caucasian pregnant women with a mean age of 30.8 ± 4.9 years (min-max: 21–39) were enrolled for the study. Most women showed a Nugent score ranging between 0 and 3 (18/24; 75%), indicating a normal lactobacilli-dominated flora. The remaining subjects were characterized by a Nugent score 4–6 (2/24; 8.3%) or ≥7 (4/24; 16.7%), indicating a progressive shift towards a condition of dysbiosis.

### Anthropometric and Nutritional Data

Anthropometric characteristics of the subjects are presented in Supplementary Table S2. No differences in BMI distribution were observed between the age groups (*p* = 0.17), and the overall trend in BMI in this study was similar when compared to the national distribution in 2019 (Statista - The Statistics Portal, 2019).

Daily intake of energy, nutrients, and alcohol are presented in Supplementary Table S3 (see Supplementary Table S4 for the distribution of intake adequacy of each dietary variable). Overall, the trend resulted was similar to other studies previously reported in the literature (Elmadfa and Freisling, 2009; Roman Viñas et al., 2011). An inadequate intake of energy from total carbohydrates and an excessive intake of protein was observed in the study population. A high intake of energy from total fat, saturated fatty acid (SFA), and sugars was evidenced in most of the women. The intake of energy from total polyunsaturated fatty acids (PUFA) and α-linolenic acid (ALA) was lower than the DRVs. Almost all the subjects did not reach the recommended goal of 25 g/day of dietary fiber. The prevalence of inadequacies was generally high for vitamins and minerals. Specifically, most of the study population did not meet the daily requirement for calcium, iron, potassium, riboflavin, folate, and vitamin D. Differently, more than two-thirds of the study population presented an adequate intake of vitamin B_6_, vitamin C, and vitamin A. Alcohol consumption was below the maximum intake level (one drink a day) for almost all the subjects.

The MEDI-LITE score ranged from 5 to 15, with most subjects having a moderate to high adherence to a Mediterranean-type diet (Supplementary Table S5). No difference in adherence distribution between the age groups (*p* = 0.16) was observed.

The mean MEDI-LITE score (10.13 ± 2.38) resulted slightly lower than the mean value for women (12.39 ± 2.39) reported in a previous publication specifically analyzing the adherence to the MD of Italian adults, using the same tool (Dinu et al., 2021).

### Correlation Between Vaginal Status and Anthropometric/Dietary Data

Significant correlations were explored between Nugent score and several anthropometric data and dietary indices (Supplementary Table S6).

**FIGURE 1:**
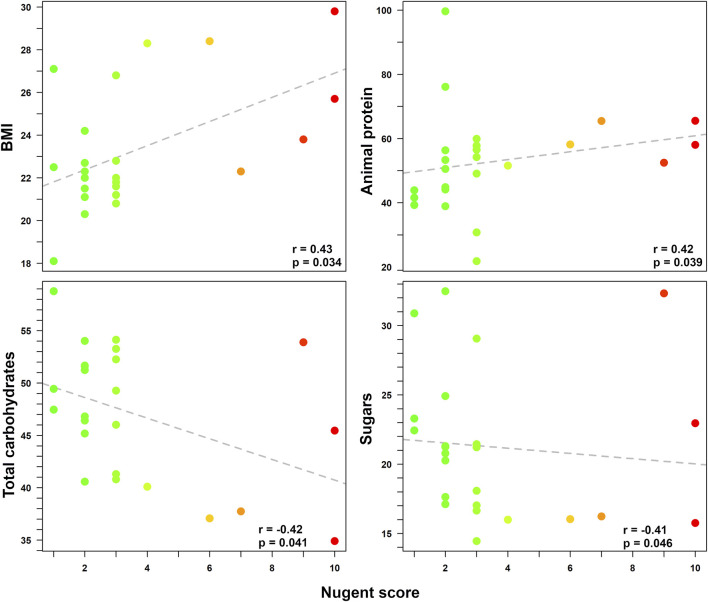
Correlations between Nugent score and various pre-pregnancy anthropometric/dietary data. Statistically significant relationships between Nugent score (0–10) and anthropometric/dietary indices (i.e., BMI, intake of animal-sourced protein, total carbohydrates, and sugars) are displayed in each box. For each correlation, Spearman coefficient (r) and *p*-value are shown. Each dot represents a woman enrolled in the study. For easier visualization, the color of the dots goes from green to red as the Nugent score increases.

A higher Nugent score (i.e., indicating a shift towards vaginal dysbiosis) was related to a higher BMI (r = 0.43; *p* = 0.034) and to a higher intake of animal-sourced protein (ASP) (r = 0.42; *p* = 0.039). Conversely, a vaginal health status (i.e., lower Nugent score) was related to a higher intake of total carbohydrates (*p* = 0.041) and sugars (*p* = 0.046). Finally, a trend between alcohol consumption and a condition of vaginal dysbiosis was also found (*p* = 0.055), even if not fully significant. The Spearman correlation coefficients (r) for the Nugent score according to the linear regressions with all anthropometric and dietary data are reported in Figure 1.

### Vaginal Metabolome

A total of 63 metabolites (mainly belonging to the groups of SCFAs, organic acids, amino acids, and biogenic amines; Supplementary Material) were detected and quantified by ^1^H-NMR spectroscopy (Supplementary Figure S1 shows portions of ^1^H-NMR spectra).

**FIGURE 2:**
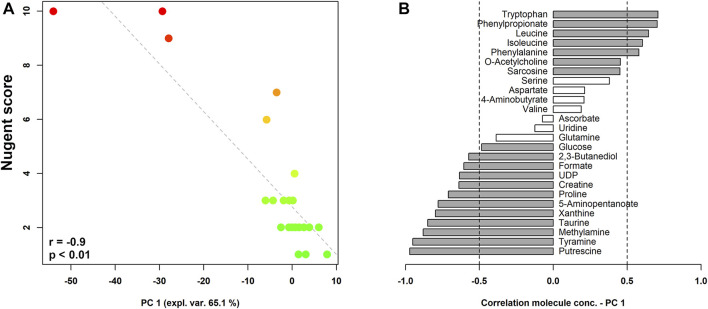
rPCA model built on the centered and scaled concentrations of the metabolites showing significant differences based on Nugent score. In the scoreplot **(A)**, the color of the dots (each representing a woman enrolled in the study) goes from green to red as the Nugent score increases. *Y*-axis shows Nugent score values. In the barplot **(B)**, describing the correlation between the concentration of each molecule and its importance over PC1, dark grey bars highlight statistically significant correlations (*p* < 0.05).

Figure 2 shows the correlation between Nugent score and the composition of the vaginal metabolome. As visualized in the correlation plot, higher levels of tryptophan, phenylpropionate, leucine, isoleucine, phenylalanine, O-acetylcholine, and sarcosine characterized the vaginal metabolome of women with a lower Nugent score (i.e., lactobacilli-dominated flora). Conversely, higher concentrations of putrescine, tyramine, methylamine, taurine, xanthine, 5-aminopentanoate, proline, creatine, UDP, formate, 2,3-butanediol, and glucose seemed to be fingerprints of women with higher Nugent scores (i.e., indicating a shift towards vaginal dysbiosis).

**FIGURE 3:**
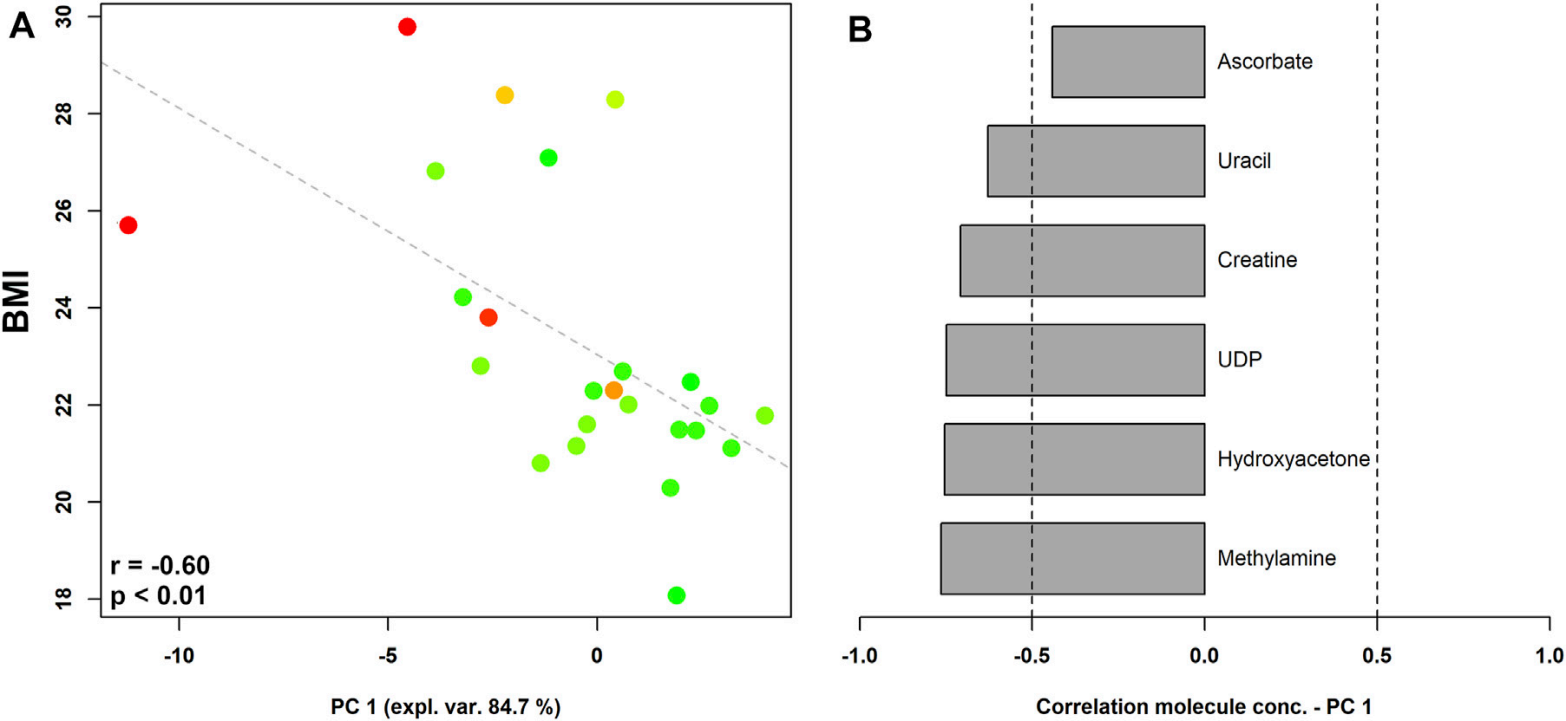
rPCA model showing correlations between pre-pregnancy BMI and vaginal metabolome in pregnant women stratified by the Nugent score. In the scoreplot **(A)**, the color of the dots (each representing a woman enrolled in the study) goes from green to red as the Nugent score increases. *Y*-axis shows the values of BMI. In the barplot **(B)**, describing the correlation between the concentration of each molecule and its importance over PC1, dark grey bars highlight statistically significant correlations (*p* < 0.05).

When pre-pregnancy BMI was correlated to vaginal metabolic profiles (Figure 3), we found that women who begun pregnancy overweight were characterized by significantly higher levels of methylamine, hydroxyacetone, UDP, creatine, uracil, and ascorbate.

**FIGURE 4:**
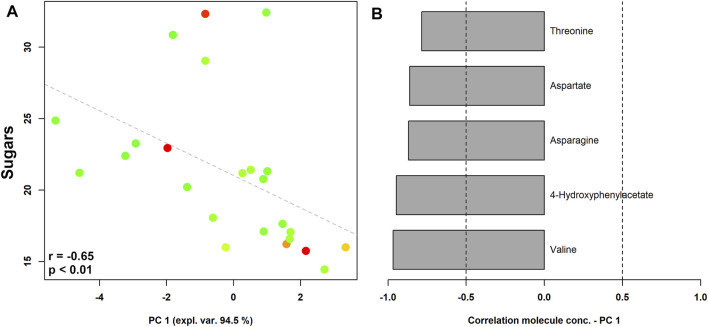
rPCA model showing correlations between pre-pregnancy sugar intake and vaginal metabolome in pregnant women stratified by the Nugent score. In the scoreplot **(A)**, the color of the dots (each representing a woman enrolled in the study) goes from green to red as the Nugent score increases. *Y*-axis shows the values of sugar intake. In the barplot **(B)**, describing the correlation between the concentration of each molecule and its importance over PC1, dark grey bars highlight statistically significant correlations (*p* < 0.05).

The vaginal metabolome of women with a higher sugar intake were characterized by higher levels of valine, 4-hydroxyphenylacetate, asparagine, aspartate, and threonine (Figure 4).

One molecule constituting vaginal metabolome, namely UDP, showed a significant negative correlation with total carbohydrate intake, whereas 5-aminopentanoate concentration was related with ASP intake (data not shown).

## Discussion

To the best of our knowledge, this is the first report evaluating the impact of pre-pregnancy anthropometric and nutritional variables on the vaginal environment of Caucasian pregnant women.

Pre-pregnancy BMI and daily energy and nutrient intake of 24 women at the first trimester of pregnancy were correlated to the bacterial (i.e., Nugent score) and metabolomic composition (i.e., ^1^H-NMR spectroscopy) of the vaginal ecosystem.

Since various factors can affect the structure of the vaginal microbiome, we excluded from the study all the women with conditions able to perturb the vaginal microbial composition (e.g., genital infections, recent use of antibiotics, heavy smoking, chronic diseases).

At first, we found that pre-pregnancy BMI was negatively related to vaginal health status, indicating that pregnant overweight/obese women have a greater occurrence of vaginal dysbiosis (i.e., BV and/or reduced number of vaginal lactobacilli).

These data agree with a previous report, showing a significant association between obesity and BV in white women (Brookheart et al., 2019). However, only a few studies have explored the relationship between pre-pregnancy BMI and BV prevalence, and a consensus on whether BMI is a risk factor for vaginal dysbiosis has not been reached.

Oh and colleagues reported that the vaginal microbiome of obese fertile women is more likely to be enriched by dysbiosis-related *Lactobacillus* species (i.e., *Lactobacillus iners*), rather than by eubiosis-associated species (i.e., *Lactobacillus crispatus*) (Oh et al., 2015).

Moreover, obesity significantly increases the diversity of the vaginal microbiota in association with *Prevotella*, an anaerobic microorganism typically found in BV-positive women (Si et al., 2017).

The mechanisms behind the association between obesity and BV are not completely understood. Presumably, disturbances in hormonal, dietary, metabolic and/or immune functions can play a significant role. Moreover, also the gut microbiota can influence the composition of the vaginal environment, acting as an extra-vaginal reservoir of BV-associated bacteria (Marrazzo et al., 2012).

When looking to the correlations between dietary data and the vaginal bacterial composition, we found that an increased risk of BV during pregnancy was associated with a higher intake of ASP.

Previous studies have mainly investigated the impact of dietary intake on the vaginal environment of American and Afro-American women, with dietary habits different from those of European Caucasian women (Neggers et al., 2007; Tohill et al., 2007; Thoma et al., 2011; Tuddenham et al., 2019).

Overall, the risk of BV has been associated with the increased dietary fat intake (Neggers et al., 2007), higher glycemic loads (Thoma et al., 2011), and lower concentrations of vitamins A, C, E, and β-carotene (Tohill et al., 2007). In addition, recently, it has been shown that diets richer in fiber are associated with lower odds of BV (Shivakoti et al., 2020).

Nevertheless, other authors failed to find associations between vaginal microbiota profiles and specific nutrient intake, including sugar, dietary fiber, protein, or fat (Song et al., 2020).

Thus, it is plausible that long-term dietary habits and energy metabolism can influence the vaginal microbiome composition, but additional large-scale studies are needed to better understand the potential role of different dietary patterns and/or specific dietary components on genital health and eubiosis.

Here, for the first time, we demonstrated that a reduced intake of ASP during the year prior to the pregnancy is crucial in maintaining a normal lactobacilli-dominated vaginal flora.

The “negative” impact of a diet rich in ASP have been previously described for the gut microbiome composition. For instance, a higher intake of plant-sourced protein is associated with greater abundance of “health-related” microorganisms in the gut (e.g., *Bifidobacterium*, *Roseburia*, *Lactobacillus*), as opposed to *Bacteroides* and *Clostridia*, found primarily in ASP (Prokopidis et al., 2020).

Similarly, it has been shown that, compared to a diet rich in ASP, diets high in plant-sourced protein are linked to a higher presence of *Bifidobacterium* in maternal milk microbiota. In turn, bifidobacteria, the hallmark of breastfed infant gut microbiota, impact positively on infant microbiota development and contributes to health outcomes in the short and long term (Cortes-Macías et al., 2021).

It is well known that the vaginal bacterial composition plays a crucial role in maternal-fetal health (Nelson et al., 2016). Healthy pregnancies are usually characterized by a lactobacilli-dominated vaginal microbiota, whereas reduced lactobacilli with increased bacterial diversity are associated with pregnancy-related complications and preterm birth (Di Simone et al., 2020).

Thus, the demonstration of an association between a pre-pregnancy excessive intake of ASP and a status of vaginal dysbiosis is of great importance, opening the way to new strategies for the prevention of negative outcomes during pregnancy.

Moreover, we found that a higher intake of total carbohydrates and sugars seemed to be associated with a condition of vaginal eubiosis (i.e., lower Nugent score, with a lactobacilli-dominated flora).

It has been hypothesized that the high starch content of the human diet can lead to high levels of glycogen in the vaginal tract, creating a suitable environment for the proliferation and dominance of lactobacilli (Miller et al., 2016; Song et al., 2020). We can therefore speculate that our results go in this direction: diets including a high intake of total carbohydrates may have led to high levels of glycogen in the vaginal tract, which, in turn, might have created a favorable environment for a lactobacilli-dominated flora (i.e., lower Nugent score). However, other studies are necessary to investigate the effect of a high carbohydrate diet on vaginal glycogen levels in humans, as well as the impact on the vaginal environment and health.

The composition of the vaginal microbiome is accompanied by specific fingerprints of the vaginal metabolome (Vitali et al., 2015; Parolin et al., 2018). In the present study, we confirmed that the vaginal environment of women with vaginal dysbiosis is characterized by higher levels of biogenic amines (e.g., tyramine, methylamine, putrescine), and organic acids (e.g., formate). Conversely, higher levels of phenylpropionate, and diverse amino acids (e.g., tryptophan, phenylalanine, isoleucine, leucine) were peculiar elements of a healthy vaginal status (Vitali et al., 2015; Ceccarani et al., 2019).

Interestingly, we found a significant association between a higher BMI and several dysbiosis-related vaginal metabolites (e.g., methylamine). Thus, this interesting interplay between BMI and vaginal metabolic profile suggests that BMI can represent a potential indicator of vaginal health.

We are fully aware of some limitations of this study: 1) the low number of women enrolled, 2) the need of more appropriate techniques to in-depth evaluate the composition of the vaginal microbiome (e.g., 16s rRNA sequencing) and metabolome (e.g., LC-MS/MS).

In conclusion, although preliminary, our study sheds light on the role of pre-pregnancy BMI and diet on the vaginal environment during pregnancy, underlining the importance of limiting protein intake from animal foods to maintain a healthy lactobacilli-dominated vaginal microbiota enriched in eubiosis-related metabolites.

Future studies are needed for a thorough comprehension of the mechanisms underlying the impact of pre-pregnancy diet on the vaginal environment to set tailored dietary approaches for the maintenance of a healthy vaginal flora during pregnancy.

## Data Availability

The original contributions presented in the study are included in the article/Supplementary Material, further inquiries can be directed to the corresponding author.
