# Supplementary material for: Pre-Pregnancy Diet and Vaginal Environment in Caucasian Pregnant Women: An Exploratory Study
Source: Front Mol Biosci. 2021 Jul 28;8:702370. doi: 10.3389/fmolb.2021.702370 (PMC8356051; doi:10.3389/fmolb.2021.702370)

Supplementary Material

Supplementary Table S1. Set of reference values used to assess energy and nutrient intakes of the study population.

Dietary variables	Dietary reference values (DRVs)	Type of DRV	Source
Energy	18–29 y: 7.9 (PAL=1.4) – 9 (PAL=1.6) MJ/day<br>30–39 y: 7.6 (PAL=1.4) – 8.7 (PAL=1.6) MJ/day	AR	(EFSA, 2017)
**Macronutrients**			
Total carbohydrates	45–60 E%	RI	(EFSA, 2017)
Sugars (monosaccharides and disaccharides)	<10 E%	RI	(WHO/FAO, 2003)
Dietary fiber	25 g/day	AI	(EFSA, 2017)
Protein	0.66 – 0.83 g per kg BW per day	AR – PRI	(EFSA, 2017)
Total fat	20–35 E%	RI	(EFSA, 2017)
Saturated fatty acids (SFA)	<10 E%	RI	(FAO, 2010)
Monounsaturated fatty acids (MUFA)	15–20 E%	RI	(FAO, 2010)
Polyunsaturated fatty acids (PUFA)	6–11 E%	RI	(FAO, 2010)
α-linolenic acid (ALA)	0.5 E%	AI	(EFSA, 2017)
Linoleic acid (LA)	4 E%	AI	(EFSA, 2017)
**Minerals**			
Calcium	18–24 y: 1000 mg/day<br>≥25 y: 950 mg/day	PRI	(EFSA, 2017)
Iron	16 mg/day	PRI	(EFSA, 2017)
Phosphorus	550 mg/day	AI	(EFSA, 2017)
Potassium	3500 mg/day	AI	(EFSA, 2017)

Sodium	2 g/day	Safe and adequate intake	(EFSA, 2017)
Zinc	LPI 600 mg/day: 9.3 mg/day	PRI	(EFSA, 2017)
**Vitamins**			
Thiamin	0.1 mg/MJ	PRI	(EFSA, 2017)
Riboflavin	1.6 mg/day	PRI	(EFSA, 2017)
Niacin	1.6 mg NE/MJ	PRI	(EFSA, 2017)
Vitamin B_6	1.6 mg/day	PRI	(EFSA, 2017)
Folate	330 µg DFE/day	PRI	(EFSA, 2017)
Vitamin C	95 mg/day	PRI	(EFSA, 2017)
Vitamin A	650 µg RE/day	PRI	(EFSA, 2017)
Vitamin E as α-tocopherol	11 mg/day	AI	(EFSA, 2017)
Vitamin D	15 µg/day	AI	(EFSA, 2017)

Abbreviations: AI, adequate intake; AR: average requirement; BW, body weight; DFE, dietary folate equivalents; DRVs, dietary reference values; E%, percentage of energy intake; LPI, level of phytate intake; MJ, megajoule (1 MJ = 238.83 kcal); NE, niacin equivalent; PAL, physical activity level (1.4 low active, sedentary lifestyle; 1.6 moderately active lifestyle); PRI, population reference intake; RE, retinol equivalents; RI, reference intake range for macronutrients; y, years.

References:

EFSA (2017). Dietary reference values for nutrients Summary report. *EFSA Supporting Publications* 14, e15121E.

FAO (2010). Fats and fatty acids in human nutrition. Report of an expert consultation. *FAO Food and Nutrition Paper* 91, 1-166.

WHO/FAO (2003). *Diet, nutrition and the prevention of chronic diseases: Report of a joint WHO/FAO expert consultation.* Geneva, Switzerland: World Health Organization.

Supplementary Table S2. Prevalence of underweight, normal weight, overweight, and obesity in the study population based on self-reported pre-pregnancy body weight and height measurements.

	**Median BMI (IQR)** kg/m^2	**Underweight** (n)	**Normal weight** (n)	**Overweight** (n)	**Obese** (n)
Age 20–29 y (n=11)	22.04 (21.15–22.40) kg/m^2	1	9	1	0
Age 30–39 y (n=13)	22.76 (21.64–26.84) kg/m^2	0	8	5	0

Abbreviations: BMI, body mass index; IQR, interquartile range; y, years.

Supplementary Table S3. Pre-pregnancy median intakes of energy, nutrients, and alcohol in the study population and their comparison to the reference values (as reported in Supplementary Table S1). Significant differences with reference values are marked in bold.

Dietary variables	Value †	*P*-value ‡
Energy	8.05 (6.73–9.72) MJ/day	0.6900
Total carbohydrates	47.14 (41.18–51.82) E%	**<0.001**
Sugars	21.00 (16.93–23.04) E%	**<0.001**
Dietary fiber	17.98 (13.79–22.16) g/day	**<0.001**
Protein	75.04 (68.94–83.31) g/day	**<0.001**
Total fat	37.75 (35.12–40.47) E%	**<0.001**
SFA	11.53 (10.34–12.35) E%	**<0.001**
MUFA	17.86 (15.53–18.75) E%	0.7400
PUFA	5.37 (4.46–6.17) E%	**<0.0001**
ALA	0.45 (0.40–0.52) E%	**0.0469**
LA	4.10 (3.53–5.04) E%	0.2864
Calcium	710.25 (575.26–832.43) mg/day	**0.0002**
Iron	10.21 (8.52–11.35) mg/day	**<0.0001**
Phosphorus	1226.31 (1100.95–1346.06) mg/day	**<0.0001**
Potassium	3146.39 (2401.40–3296.50) mg/day	**<0.001**
Sodium	2088.30 (1894.28–2372.93) mg/day	0.3600
Zinc	9.86 (8.71–10.84) mg/day	0.4056
Thiamin	0.93 (0.83–1.24) mg/day	**0.0255**
Riboflavin	1.51 (1.34–1.65) mg/day	0.1400
Niacin	20.39 (16.19–25.35) mg NE/day	**<0.0001**
Vitamin B_6	1.92 (1.52–2.24) mg/day	**0.0027**
Folate	262.18 (209.69–297.40) µg DFE/day	**<0.001**
Vitamin C	113.42 (86.81–142.21) mg/day	**0.0300**
Vitamin A	794.96 (628.47–920.25) µg RE/day	**0.006**
Vitamin E	12.43 (10.53–15.80) mg/day	**0.0269**
Vitamin D	2.66 (2.34–3.90) µg/day	**<0.001**
Alcohol	3.64 (0.95–9.94) g/day	**0.0366**

Abbreviations: ALA, α-linolenic acid; DFE, dietary folate equivalents; E%, percentage of energy intake; LA, linoleic acid; MUFA, monounsaturated fatty acids; NE, niacin equivalent; PUFA, polyunsaturated fatty acids; RE, retinol equivalents; SFA, saturated fatty acids.

[†] median with interquartile range (Q1 to Q3).

[‡] p values from Student's t-test for normally distributed variables (energy, total carbohydrates, sugars, dietary fiber, protein, total fat, SFA, MUFA, ALA, iron, potassium, sodium, zinc, thiamin, niacin, vitamin B_6, folate, vitamin C, vitamin D) and Mann-Whitney U test for non-normally distributed variables (PUFA, LA, calcium, phosphorus, riboflavin, vitamin A, vitamin E, alcohol).

Supplementary Table S4. Pre-pregnancy adequacy of dietary intake of the study population in comparison to the reference values.

Dietary variables	Adequate intake n (%)	Low intake n (%)	Excessive intake n (%)
Energy	6 (25%)	10 (42%)	8 (33%)
Total carbohydrates	17 (71%)	7 (29%)	0 (0%)
Sugars	0 (0%)	--	24 (100%)
Dietary fiber	3 (13%)	21 (88%)	--
Protein	3 (13%)	0 (0%)	21 (88%)
Total fat	6 (25%)	0 (0%)	18 (75%)
SFA	5 (21%)	0 (0%)	19 (79%)
MUFA	17 (71%)	5 (21%)	2 (8%)
PUFA	7 (29%)	17 (71%)	0 (0%)
ALA	8 (33%)	16 (67%)	0 (0%)
LA	13 (54%)	11 (46%)	0 (0%)
Calcium	2 (8%)	22 (92%)	--
Iron	0 (0%)	24 (100%)	--
Phosphorus	24 (100%)	0 (0%)	--
Potassium	1 (4%)	23 (96%)	--
Sodium	10 (42%)	--	14 (58%)
Zinc	14 (58%)	10 (42%)	--
Thiamin	15 (63%)	9 (38%)	--
Riboflavin	8 (33%)	16 (67%)	--
Niacin	24 (100%)	0 (0%)	--
Vitamin B_6	16 (67%)	8 (33%)	--
Folate	3 (13%)	21 (88%)	--
Vitamin C	17 (71%)	7 (29%)	--
Vitamin A	17 (71%)	7 (29%)	--
Vitamin E	15 (63%)	9 (38%)	--
Vitamin D	0 (0%)	24 (100%)	--
Alcohol	19 (79%)	--	5 (21%)

Supplementary Table S5. Prevalence of low, moderate, and high adherence to a Mediterranean-type diet before pregnancy in the study population.

	**Median MEDI-LITE score (IQR)**	**Low adherence** (n) †	**Moderate adherence** (n) †	**High adherence** (n) †
Age 20–29 y (n=11)	10.00 (9.50–11.50)	2	8	1
Age 30–39 y (n=13)	10.00 (8.00–13.00)	0	9	4

Abbreviations: IQR, interquartile range; y, years.

† Low adherence range 0–6; moderate adherence range 7–12; high adherence range 13–18.

Supplementary Table S6. List of the main pre-pregnancy anthropometric and dietary data for which a correlation with vaginal health status (Nugent score) during pregnancy was investigated. Correlations were searched by calculating Spearman correlation coefficient (r) after correction for multiple comparisons (i.e., Bonferroni-Holm correction). Significant differences with reference values are marked in bold.

**Parameter**	**r**	***P-value***
**BMI**	**0.43**	**0.034**
**Animal protein**	**0.42**	**0.039**
**Total carbohydrates**	**-0.42**	**0.041**
**Sugars**	**-0.41**	**0.046**
Alcohol	0.40	0.055
Calcium	0.38	0.068
Riboflavin	0.36	0.083
Phosphorus	0.34	0.105
MUFA	0.32	0.133
Total fat	0.30	0.160
Carotene	0.28	0.188
Total protein	0.26	0.215
PUFA	-0.26	0.221
LA	-0.26	0.226
Zinc	0.23	0.279
Vitamin D	-0.18	0.402
Potassium	0.16	0.452
Vitamin B_6	0.16	0.457
Oleic acid	0.16	0.464
Cholesterol	-0.15	0.484
Folate	0.14	0.507
Niacin	0.14	0.508
Dietary fiber	0.14	0.525
Iron	0.14	0.525
Vitamin C	0.12	0.580
ALA	0.10	0.630
SFA	0.09	0.676
Vitamin A	0.08	0.693
Thiamin	0.08	0.694
Vitamin E	0.05	0.822
Sodium	0.04	0.851
Plant protein	0.04	0.861
Energy	0.02	0.937
MEDI-LITE score	-0.01	0.952

Supplementary Figure S1. Portions of ^{1}H-NMR spectra, representative of all the spectra obtained in this study. The name of each molecule appears over the signal employed to quantify it. To optimize the visualization of each portion, a different spectrum with a convenient signal-to-noise ratio was selected.

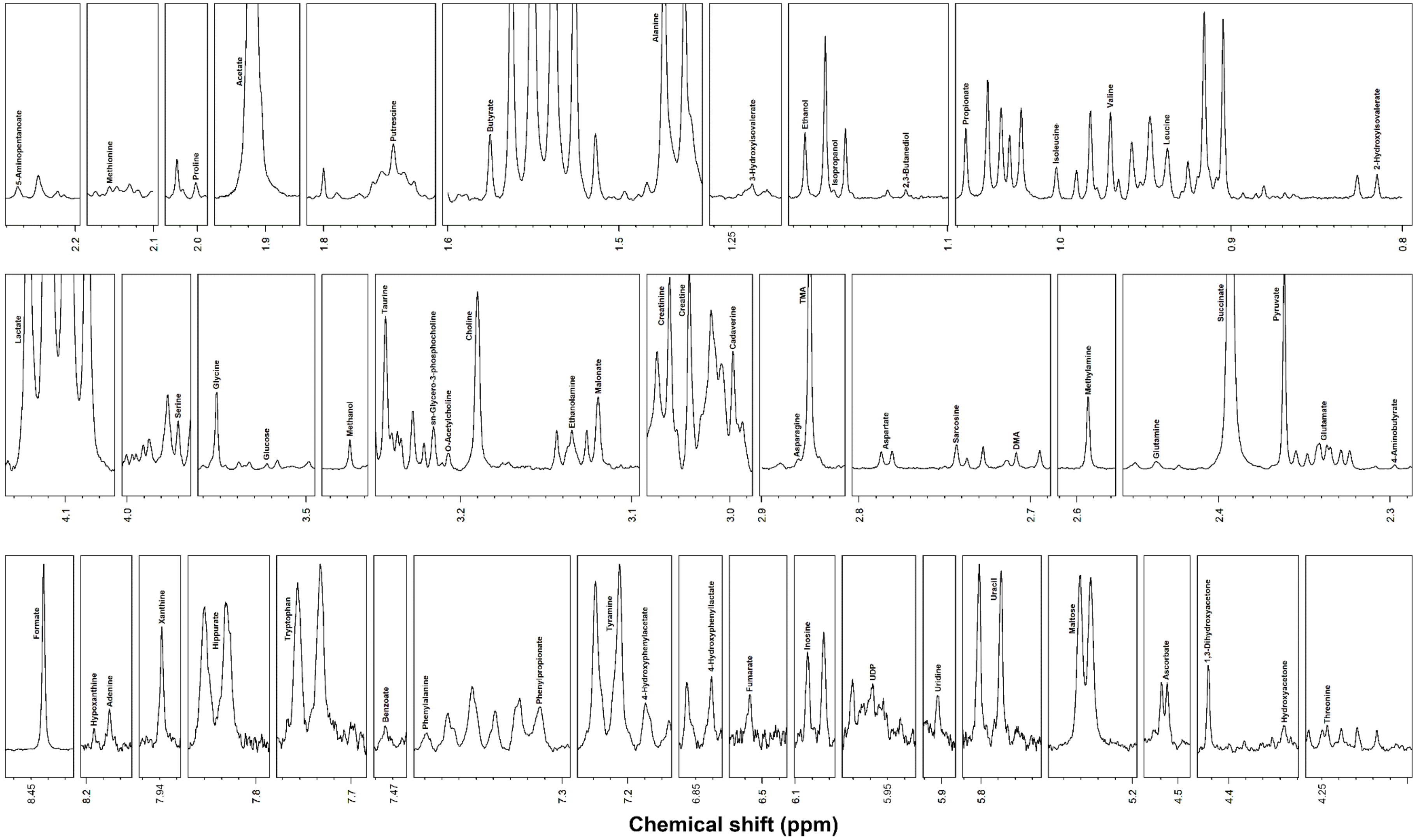

Supplement: Supplementary file 1 [file DataSheet1.pdf]
